# Cross-Cultural Adaptation of the Instrument “Nurse–Physician Relationship Survey: Impact of Disruptive Behavior in Patient Care” to the Spanish Context

**DOI:** 10.3390/healthcare10101834

**Published:** 2022-09-22

**Authors:** Pedro Moreno-Leal, César Leal-Costa, José Luis Díaz-Agea, Ismael Jiménez-Ruiz, María Suarez-Cortés, Adriana Catarina De Souza Oliveira

**Affiliations:** 1 Faculty of Nursing, Universidad Católica de Murcia, 30107 Murcia, Spain; 2 Faculty of Nursing, Universidad de Murcia, 30120 Murcia, Spain

**Keywords:** problem behavior, patient safety, physician–nurse relations, cultural competency, methodological studies

## Abstract

Disruptive behavior in the healthcare context has an impact on patient care, healthcare personnel, and the health organization, and it also influences the therapeutic relationship, communication process, and adverse events. However, there is a lack of instruments that could be used for its analysis in the hospital care environment in the Spanish context. The objective of the study was to culturally adapt and perform a content validation of the tool “Nurse–Physician Relationship Survey: Impact of Disruptive Behavior on Patient Care”, to the Spanish content (Spain). An instrumental study was conducted, which included an analysis of conceptual and semantic equivalence. A panel of experts analyzed the translations, by analyzing the Content Validity Index (CVI) of the group of items in the scale through the Relevance Index (RI) and the Pertinence Index (PI). Only a single item obtained an RI value of 0.72, although with PI value of 0.81, with consensus reached for not deleting this item. The CVI of all the items was >0.80 for the mean value of the RI, as well as the PI. The instrument was adapted to the Spanish context and is adequate for evaluating the disruptive behaviors on nurse–physician relationships and its impact on patient care. However, the importance of continuing the analysis of the rest of the psychometric properties in future studies is underlined.

## 1. Introduction

Disruptive behavior is defined as any inadequate behavior, confrontation, or conflict—ranging from verbal abuse (abusive, intimidating, disrespectful, or threatening behavior), to physical or sexual abuse—that could negatively affect work relations, communication efficiency, and the caregiving process and its results [1,2,3].

In the healthcare context, the relationship between disruptive behavior and the negative consequences for the patient, for the healthcare personnel, and for the organization have been demonstrated, having an influence on the therapeutic relationship, communication process, and adverse events and dissatisfaction and compromised patient safety [4,5,6,7,8,9,10]. Moreover, Keller et al. [5] stressed that at the organizational level, the physical and emotional workload in services with a high variety of processes, can be a predictor of disruptive behaviors. For example, if communication is incorrect, or if there is some type of disruptive behavior, there is a greater probability that a mistake in care can be produced [11]. In 2006, the Joint Commission International (JCAHO) named disruptive behavior as a Sentinel Event Alert 40 due to its deconstructive effect on the culture of safety [6].

The study conducted by Rosenstein et al. [3] to analyze the disruptive behavior with respect to the nurse–physician relationship and its impact on patient care, is considered an important reference for addressing disruptive behaviors in area of healthcare. In this specific case, through the application of the original instrument used in our transcultural adaptation, the results showed that 53% of the health professionals identified the disruptive behaviors as predictors of adverse events.

A systematic review has recently been carried out to provide the best methodological quality scientific evidence available on disruptive behavior at a hospital, the aspect associated with the safety of the patient, and its impact on quality of care [12]. Of the studies identified in the systematic review, many measured disruptive behaviors in specific samples of professional groups such as physicians [13] or nurses [14,15], in specific clinical areas such as the surgical setting [16]. Other studies measured other variables with different instruments [17,18,19]. Only two studies analyzed this problem from a multidisciplinary perspective, and both used the Rosenstein instrument [3,20]. 

In the Spanish context, we did not find any instruments on the subject. Thus, our aim was to create an instrument that is adequate to the language and context of Spain. In the preparatory phase, as a step prior to adapting the instrument, a systematic review was conducted to discover the current state of the subject and instruments in this area [12]. We decided to adapt the instrument “Nurse–Physician Relationship Survey: Impact of disruptive behavior on patient care” [1], which was originally composed of 22 items. Of these, 17 items (Likert-type scale and dichotomous questions) are related to: (a) frequency of physician and nurse disruptive behavior (nine items), (b) importance of physician–nurse relationship and its effects on patient care and safety (four items), (c) reporting disruptive behaviors (one item), (d) clinical and psychological effects of disruptive behaviors (two items), and e) adverse events of disruptive behaviors (1 item). In addition, there is an open section for comments and four social-labor variables. 

The objective of the study was to transculturally-adapt the instrument “Nurse–Physician Relationship Survey: Impact of disruptive behavior on patient care” to the Spanish versions (Spain), and to validate the content with a panel of experts.

## 2. Materials and Methods

Instrumental study of transcultural adaptation and validation of content of an instrument to the Spanish version (Spain). 

**Figure 1:**
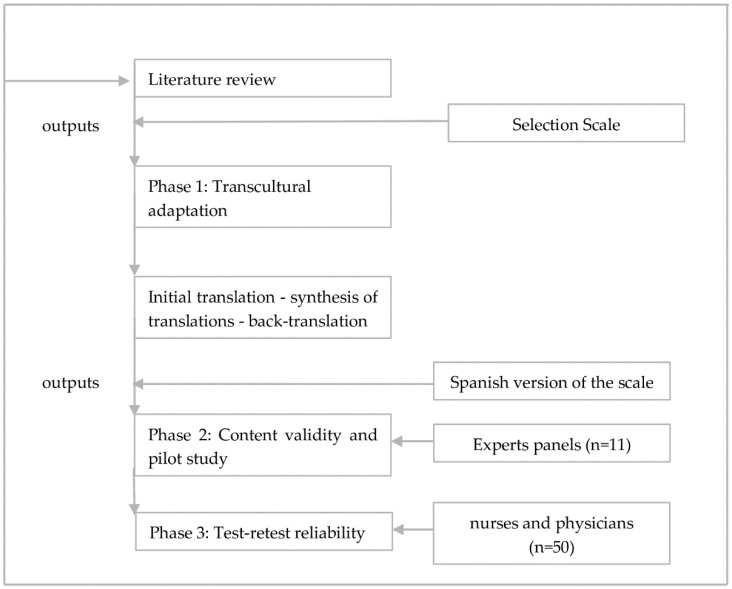
Phases of the study.

For the methodological process of the study, 3 phases were implemented, following international guidelines [21]: First Phase: Transcultural adaptation (initial translation, synthesis of the translations, back-translation) [22]. Second Phase: Analysis of content validity through a panel of experts and pilot study. Third Phase: Test–retest reliability (Figure 1).

### 2.1. Phase 1: Transcultural Adaptation

For the initial translation, a translation was made from English to Spanish (Spain) by three bilingual translators (mother tongue Spanish/Spain, with English language certification), independently. One of them knew the objective of the study, and a request was made that the translation had a semantic perspective (semantic equivalence to the Spanish context—without adding any new concept) [22,23]. 

In this phase, the three translators were asked to indicate dubious words and their corresponding suggested changes, and if the question was well formulated.

The stage of back-translation from Spanish (Spain) to English was performed by three bilingual translators (mother tongue English with a certification for the Spanish (Spain) language), independently, and began after the synthesis of the translations from the first stage.

### 2.2. Phase 2: Analysis of the Content Validity through a Panel of Experts and Pilot Study

For this phase, an adaptation of the conventional Delphi technique was utilized [24,25]. To provide a response to this analysis, a panel of experts was composed by utilizing two strategies. On the one hand, to recruit the experts, a search for information was conducted with managers in healthcare (titles, functions, years of service, participation in panels in related areas), who were identified with the help of databases of scientific evidence published.

After these steps, 28 health professionals were invited (nurses and/or physicians) by phone. An explanation was given as to the object of study, and they were told that participation was on an individual basis, without information provided on the composition of the panel [25]. Moreover, they were informed that that the confidentiality and protection of data were guaranteed, according to the Organic Law 3/2018, from 5 December, of Protection of Personal Data and guarantee of digital rights [26]. 

The inclusion criteria were (a) ≥5 years of experience in hospitals (professionals from different units and different hospitals), (b) having previously participated in other research studies or publication in patient safety (associated in some manner to the subject under study: patient safety, disruptive behavior, and/or communication between health professionals), (d) capacity to provide opinions and suggestions, and (e) motivation and availability to participate in the study.

To evaluate the suitability of the panel of experts, the Competence Coefficient (K), represented by (K) = 0.5 (Kc + Ka), was calculated, with (Kc) being the Knowledge Coefficient, defined as the self-evaluation of the experts about their knowledge with respect to the subject studied, and (Ka) the Argumentation Coefficient, defined the arguments (sources) used to prove their knowledge [27]. Table 1 shows the score applied for Ka. 

After evaluating the value of the Coefficient of Competence (K), 11 experts were selected [28], who were provided with the translated versions of the scale for them to judge each of the items and the scale in general. This number of experts was considered sufficient for evaluating the content validity [27,29].

This evaluation was performed in two rounds. In the first round, the experts evaluated if the item was clear and comprehensible (consensus from at least 70% of the judges) [30]. In the second round, they evaluated the Relevance Index (RI), and the Pertinence Index (PI) of the items of the scale, with 0 = not pertinent/not relevant, and 1 = pertinent/relevant. The formula for its calculation was Number of experts who scored with a 1 divided by the total number of experts who evaluated the item. Lastly, the Content Validity Index (CVI) of the entire set of items of the scale was calculated (mean of the RI/PI of the items as a set). A value of ≥8 was considered as an accepted validity for each of the items, as well as for the Scale as a whole [28,30]. In this phase, the qualitative observations of the experts were collected, for each of the items that shaped the original instrument. 

A pilot study was conducted with 10 nurses. The objective was to evaluate is understandability, acceptability, and time for completion of the scale for the evaluators.

### 2.3. Phase 3: Test–Retest Reliability

Finally, the test–retest reliability was analyzed using the Kappa index [31], the Intraclass Correlation Coefficient (ICC) [32] and the general concordance index according to the nature of the variable in a sample of 50 participants (nurses and physicians) from different hospital units/services, carrying out clinical or management activities. It has been applied in an interval of 15 days between test and retest.

As for the ethical aspects, the ethics and copyright principles were followed, with authorization solicited from Alan H. Rosenstein (the main author of the original instrument), and a commitment was made to provide information on the phases of the study and the evaluation of the final adapted version (before its publication).

## 3. Results

After the process of translation, the three translators created an online version where they indicated the dubious words. So that the best transcultural version was selected, the panel of experts, after analyzing the items in which these words were found, created a matrix of composition of the items, according to the analysis of clarity and comprehension of these items, and their cultural and idiomatic semantics (Table 2). The panel of experts stressed the importance of adding the definition “disruptive behavior” at the start of the Scale (before the first question).

Table 3 shows that for the most part, the experts obtained a high Coefficient of Competences (K), with values 0.8 < K < 0.9. However, one of them (expert 4) was excluded from the panel for not answering the item Argumentation coefficient (Ka), so that the composition was finally comprised by 11 experts.

Table 4 shows the composition of the items after the content validity analysis. For the RI, the only item that obtained an RI value < 0.8 was item 5, “Is there a specialization in which disruptive behaviors are produced frequently?” being susceptible for elimination. However, this item obtained a PI of 0.81, and a consensus was made to not delete the item, according to the evaluation of the suggestions and opinions of the experts and study researchers. No items were eliminated or added with respect to the original instrument. Likewise, no changes were made to the response scales of the items. The CVI calculated for the complete scale was RI = 0.89, and PI = 0.94.

After the cognitive pilot study, it was verified that all the nurses understood the writing and sense of the items. The questionnaire completion time was 10 min.

The sociodemographic and professional characteristics of the 50 participants used to measure temporal stability (test–retest reliability) were women (60%) with a mean age of 42 (SD = 11.6) years, 52% being physicians, and 74% of the participants performing their care work in the clinical setting.

As we can see in Table 4, most of the items presented a Kappa Index with values that indicated a concordance of the responses between moderate and almost perfect (16 items), ICC with values equal to or greater than 0.75 (indicating a reproducibility excellent) and general agreement indices of 100% in all items (Table 4).

As the result of the study, a version that was transculturally adapted to Spanish (Spain) was created, given the consensus by the panel composed of 11 experts (Appendix A, Table A1).

## 4. Discussion

Like in other studies investigating the disruptive behavior [4,5,6,7,8,9,10], our results indicate that, after translating and adapting the “Nurse–Physician Relationship Survey: Impact of disruptive behavior on patient care” [1] to Spanish, the tool is a valid, ready to be used in Spain, and opening possibilities to study disruptive behaviors in Spain and compare the results internationally. 

After a systematic review [12] on disruptive behavior, it was discovered that no studies existed in Spain on the construction or transcultural adaptation of an instrument on disruptive behavior in healthcare, and its impact on safe care. However, when the instrument “Nurse–Physician Relationship Survey: Impact of disruptive behavior on patient care” [1] was identified, it was observed that its approach could provide an answer to our questions, to identify aspects related with disruptive behaviors in healthcare, and its association to adverse events, satisfaction, communication, and improvement strategies. Moreover, we considered the international standing and prestige of the authors of the original version with respect to the subject studied, with this questionnaire used in various studies and in different specializations [2,3,4,5,6,7,8,9,10,20]. None of the studies found carried out a cross-cultural adaptation with the methodological rigor used in our study, and none of them carried out a reliability study. This makes it difficult to compare the results of our study with other similar studies. This justified our study, as it was agreed that a process of adaptation would be more efficient instead of the creation of a new instrument, which would be more time-consuming and costly. 

The process of transcultural adaptation in the healthcare environment dates to the 1960s in mental health. However, perhaps with a different process of adaptation with “less rigor” and specificity for each instrument, given the scientific and methodological advancements in that period [33]. Presently, the process of cultural adaptation consists of a rigorous methodological process that is not solely based on linguistic translation. Steps are taken to achieve a high quality linguistic and transcultural version. The method by Beaton et al. [22] utilized in the present study, is considered one of the most utilized for transcultural adaptation, as it recommends a rigorous follow-up and monitoring of systematized stages and the participation of the author of the original questionnaire. In our case, the author (original version), aside from authorizing the use of the instrument, also monitored the stages of the study and approved the final version created by the panel of experts. 

It was not easy to define the methodological process of adaptation and validation, given the variability of the response scales. However, the high Competence coefficient (K) obtained by the panel of experts ensured an adequate level of competence when evaluating the instrument [27]. It is important for an expert to be familiar with the area studied, and to possess a good level of knowledge to evaluate a guide or protocol [20].

With respect to the composition of the items, all the items, or dubious words found in the translation process obtained a 95% consensus from the panel of Experts, justifying every decision. An aspect that must be underlined was the “resounding” decision of the panel of experts with respect to the reporting of adverse events, indicating the exclusion of the term “non-punitive”, as it is inherent and intrinsic to a reporting system. Effectively, the evidence shows that confidentiality, non-punitive actions, and anonymity, are basic principles of a reporting system, which at the same time, improves the degree of safety of the patient and the quality of care, creating a positive and collaborative environment, one of non-rejection and under reporting [34,35].

The only item that obtained an RI < 0.80 was item 5 “Are there any particular specialties where disruptive events occur most?” which was not eliminated after the evaluation by the panel of experts and researchers. The justification was that this item could not be substituted by or be part of item 4, as it was understood that it is not only important to identify the setting (unit), but also to discover if there is a more prevalent specialties with respect to disruptive behavior. Moreover, this item obtained a PI of 0.81, whose value indicated that the item was appropriate, opportune, and convenient for the subject proposed. The PI of the items is their degree of pertinence with respect to the object of study and the research objectives as well [36]. The results have shown that the items have an adequate content validity, which ensures that the items from the instrument are adequate and a representative sample of the content to be evaluated [29]. 

The Delphi method used in the study allowed the instrument adaptation proposal to be subjected to the judgement of experts, who assessed and evaluated the quality criteria necessary for the instrument to be a consistent and coherent scale, and that the concepts explored from another culture could be significantly reproduced to the culture found in the healthcare system in the Spanish context.

According to Kyle et al. [37], organizations do not understand how disruptive behaviors appear in the healthcare context, or how they can be measured, as there is little research on the subject. For this reason, stress is placed on the importance of conducting studies that identify disruptive behaviors and that analyze improvement strategies, to promote a culture that is proactive in the prevention and monitoring of these behaviors. 

This study is not without limitations. The use of a sample of 11 experts, 10 nurses and 50 physicians/nurses could create possible bias with respect to the subjectivity and generalization of the results. However, carrying out the three phases of the study, with the different samples, gave the study methodological rigor. Another limitation could be that the pilot was only carried out with nurses. Lastly, one more limitation could be that in the third phase of the study some relevant background of the respondents (public, rural, urban hospitals, etc.) were not collected.

## 5. Conclusions

The instrument Nurse–Physician Relationship Survey: Impact of disruptive behavior on patient care was adapted to the Spanish context and is adequate for evaluating the disruptive behaviors on nurse–physician relationships and its impact on patient care. The use of a systematic and rigorous methodology allowed us to obtain a conceptual version that is linguistically equivalent to the original. 

The clinical applicability of the instrument to measure disruptive behavior in the relationships of health professionals would be to identify the factors associated with said behaviors and their impact on health care. The evaluation of disruptive behaviors can be used to design prevention strategies and improve the quality of care and patient safety. Nevertheless, the importance of continuing with the analysis of the rest of the psychometric properties in future studies is underlined.

## Figures and Tables

**Table 1:** Scores of the sources of argumentation to obtaining the “Argumentation coefficient” (Ka).

Source of Argumentation	High	Medium	Low
Theoretical analysis who have developed related with the subject studied	0.3	0.2	0.1
Your professional experience	0.5	0.4	0.2
Review of research studies on the subject with Spanish/national authors	0.05	0.05	0.05
Review of research studies on the subject with non-Spanish/international authors	0.05	0.05	0.05
Your own knowledge on the current state of the subject	0.05	0.05	0.05
Your intuition about the study/subject	0.05	0.05	0.05

Adapted from Cabero and Barroso [27].

**Table 2:** Matrix of composition of the final items according to the panel of experts.

Original	Translator 1	Translator 2	Translator 3	Panel of Experts	Decision from Panel of Experts—Composition of the Item
Survey on the relationship Nurse—Physician: Impact of disruptive behavior in patient care	Encuesta sobre la relación Enfermera—médico: impacto del comportamiento disruptivo en la atención al paciente	Escala sobre la relación Enfermera—médico: impacto del comportamiento disruptivo en la atención al paciente	Cuestionario sobre la relación Enfermera—médico: impacto del comportamiento disruptivo en la atención al paciente	Escala sobre la relación Enfermero/a—médico: impacto del comportamiento disruptivo en la atención al paciente (Scale on the relationship Nurse–physician: impact of disruptive behavior on patient care)	Scale on the relationship Nurse–physician: impact of disruptive behavior on patient care. Justification: Although a consensus of 70% of the translators was not found, a consensus of 95% was obtained from the Panel of Experts, who decided to name the instrument as a SCALE, considering the non-grouping of the items according to dimensions or categories.
In every item: disruptive behavior	Comportamiento inapropriado	Comportamiento disruptivo	Comportamiento perturbador/disruptivo	Disruptive behavior adding the definition at the beginning of the Scale (before the first question).	“Disruptive behavior” was defined as any inappropriate behavior, altercation or conflict ranging from verbal abuse to physical or sexual abuse. One of the potential consequences of disruptive behavior is its effect on the collaboration and communication between physicians and nurses, which can lead to negative results in patient care. The present questionnaire was designed to identify the potential impact of disruptive behavior on adverse events, medical mistakes, patient safety, quality, and other aspects associated to care. Justification: There is a 100% consensus between the experts, who selected the term “disruptive behavior” considering the reference studies, including the original study on the instrument and the analysis of its concept. Moreover, an indication was made to add all the information before the first question (concept of disruptive behavior, objectives, and indications).
In every item: **physicians and nurses**	Médicos y enfermeros	Médicos y enfermeros/as	Médicos y enfermeros	Médicos y enfermeros/as	Justification: Although a consensus of 70% was not found between the translators, a 95% consensus was found in the panel of experts, who decided to identify the nursing category with the translation “enfermeros/as”, as in Spanish, the ending “os” and “as” refer to male or female nurses, respectively. Moreover, this is the idiomatic expression used in healthcare. However, in the physicians category, the word “medicos” is maintained, independently of the sex of the healthcare professional.
Item 4. Are there any **particular settings where** disruptive behavior is most prevalent?	Zona o lugar en concreto	Unidad en concreto	Unidad en concreto	Unidad en concreto	¿Hay alguna unidad en concreto en la cual el comportamiento disruptivo sea más frecuente? (Are there any particular settings where disruptive behavior is most prevalent?) Justification: there was a 95% consensus in the panel of experts leading to the decision of using the term “unidad” (unit), because the term “zona” (*area*) is a limited space that is most frequently used in geography or public administrations. On the other hand, the term “lugar” (*place*) can create confusion in its interpretation, for example lugar-puesto (*place-position*), lugar-cargo (*place-load*). In the Spanish context, it is more frequent to use “unidad” to identify a physical space, where at the level of hospitals, we find equipment and personnel for the monitoring and treatment of patients.
Item 8. **How often does** physician disruptive behavior occur at your hospital?	¿Con qué frecuencia…?	¿Cuál es la frecuencia…?	¿Con qué frecuencia…?	¿Con qué frecuencia…?	¿Con qué frecuencia los médicos presentan comportamientos disruptivos en su hospital? (*How often does physician disruptive behavior occur at your hospital?)* Justification: There was a 100% consensus from the experts who selected “¿con qué frecuencia? (*how frequently*), because it was understood that it was the most semantically-adequate term.
Item 9. **How often does** nurse disruptive behavior occur at your hospital?	¿Con qué frecuencia…?	¿Cuál es la frecuencia…?	¿Con qué frecuencia…?	¿Cuál es la frecuencia…?	¿Con qué frecuencia los/as enfermeros/as presentan comportamientos disruptivos en su hospital? (*How often does nurse disruptive behavior occur at your hospital?*) Justification: There was a 90% consensus from the experts who decided that selected “¿con qué frecuencia? (*how frequently*), was the most semantically-adequate term.
Item 15: **Are you aware of** any potential adverse events that could have occurred from disruptive behavior?	Tiene conocimiento	Estar al tanto	Tiene conocimiento	Tiene conocimiento	¿Tiene conocimiento de cualquier evento adverso potencial que pudiera haber ocurrido debido un comportamiento disruptivo? (*Are you aware of any potential adverse events that could have occurred from disruptive behavior?*) Justification: There was a 95% agreement that “¿tiene conocimiento?” (*do you have knowledge on*) was the most semantically-adequate for a scientific instrument.
Item 20. Is there a **non-punitive reporting environment** for those who witness /experience disruptive behavior?	¿Existe un sistema de notificación para los eventos presenciados o cometidos por los médicos y enfermeros/as?	¿Existe un entorno de registro no punitivo para aquellos que presencian / experimentan comportamiento disruptivo?	¿Existe un sistema de notificación para los eventos presenciados o ocasionados por los médicos y enfermeros/as?	¿Existe un sistema de notificación para los eventos presenciados o cometidos por los médicos y enfermeros/as?	¿Existe un sistema de notificación para los eventos presenciados o cometidos por los médicos y enfermeros/as? (*Is there a non-punitive reporting environment for the events witnessed or committed by physicians and/or nurses*) Justification: There was a 100% agreement that this was the correct translation, considering all the international recommendations what indicate that healthcare institutions/organizations must implement a system of reporting that is inherent to a non-punitive culture and which adheres to the confidentiality of the personal data of those who report.

**Table 3:** Result of the Competence Coefficient (K) of the panel of Experts.

Expert	Knowledge or Information Coefficient (Kc) = nc * (0.1)	Argumentation Coefficient (Ka) = fa * 1 + fa * 2…. + fa * 6	Competence Coefficient (K) = 0.5 (Kc + Ka)	Panel of Experts
Expert 1	0.8	0.9	0.85	Included—K high *
Expert 2	0.9	0.9	0.9	Included—K high *
Expert 3	0.7	0.7	0.7	Included—K medium *
Expert 4	0.5	NA *	NA *	Excluded—no value for K
Expert 5	0.7	0.8	0.75	Included—K medium *
Expert 6	0.8	0.9	0.85	Included—K high *
Expert 7	0.9	0.9	0.9	Included—K high *
Expert 8	0.9	0.9	0.9	Included—K high *
Expert 9	0.7	0.8	0.75	Included—K medium *
Expert 10	0.8	0.9	0.85	Included—K high *
Expert 11	0.7	0.7	0.7	Included—K medium *
Expert 12	0.8	0.8	0.8	Included—K high *

nc * = self-evaluation of the experts about their level of knowledge, Scale from 1 to 10; fa * = sources of argumentation for items 1 to 6; NA * = No answered; K high * = 0.8 < K < 1.0; K medium * = 0.5 < K < 0.8.

**Table 4:** Relevance Index (RI) and Pertinence Index (PI) of the items of the scale.

Item	Composition of the Item	RI	PI	RS
1	En una escala de 1 a 10, siendo 10 la respuesta más positiva ¿Cómo describiría el ambiente de la relación Enfermero/a—Médico en su hospital? (On a scale of 1—10 with 10 being the most positive, how would you describe the overall atmosphere of nurse–physician relationships at your hospital*?*)	0.81	0.90	0.859 ^a^
2	¿Alguna vez ha presenciado comportamiento disruptivo por parte de un/a médico de su hospital? (Have you ever witnessed disruptive behavior from a physician at your hospital?)	0.90	0.90	0.552 ^b^
3	¿Alguna vez ha presenciado comportamiento disruptivo por parte de un/a enfermero/a de su hospital? (Have you ever witnessed disruptive behavior from a nurse at your hospital?)	0.90	0.90	0.425 ^b^
4	¿Existe alguna unidad en concreto en la cual el comportamiento disruptivo es más prevalente? (Are there any particular settings where disruptive behavior is most prevalent?)	0.81	1	100 ^c^
5	¿Existe alguna especialidad donde se produzcan comportamientos disruptivos de forma frecuente? (Are there any particular specialties where disruptive events occur most?)	0.72	0.81	100 ^c^
6	¿Qué porcentaje de médicos diría que muestran comportamiento disruptivo en su hospital? (What percentage of physicians would you say exhibit disruptive behavior at your hospital?)	0.81	0.90	0.807 ^b^
7	¿Qué porcentaje de enfermeros/as diría que muestran comportamiento disruptivo en su hospital? (What percentage of nurses would you say exhibit disruptive behavior at your hospital?)	0.81	1	0.834 ^b^
8	¿Con qué frecuencia los médicos presentan comportamientos disruptivos en su hospital? (How often does physician disruptive behavior occur at your hospital?)	0.81	1	0.457 ^b^
9	¿Con qué frecuencia los enfermeros/as presentan comportamientos disruptivos en su hospital? (How often does nurse disruptive behavior occur at your hospital?)	0.81	1	0.726 ^b^
10	¿Cómo de graves son los problemas causados por el comportamiento disruptivo de los médicos en su hospital? (how serious of an issue is physician disruptive be-havior at your hospital?)	1	1	0.895 ^a^
11	¿Cómo de graves son los problemas causados por el comportamiento disruptivo de enfermeros/as en su hospital? (How serious of an issue is nurse disruptive behavior in your hospital?)	1	1	0.909 ^a^
12	¿Cree que el comportamiento disruptivo puede tener efectos potencialmente negativos en la atención al paciente? (Do you think that disruptive behavior can potentially have a negative effect on patient outcomes?)	1	1	1 ^b^
13	¿Con qué frecuencia cree que el comportamiento disruptivo influye en los siguientes aspectos? (How often do you think disruptive behavior results in the following?)	1	1	0.737 ^b^
14	¿Con qué frecuencia considera que existe una relación entre el comportamiento disruptivo y los siguientes aspectos? (How often do you think there is a link between disruptive behavior and the following?)	1	1	0.835 ^b^
15	¿Conoce cualquier evento potencialmente desagradable que puede haber ocurrido debido al comportamiento disruptivo? (Are you aware of any potential adverse events that could have occurred from disruptive behavior?)	0.81	1	1 ^b^
16	Si la respuesta es sí, ¿Cómo de grave hubiera sido el impacto en los pacientes? (If yes, how serious an impact do you think this could have had on patient outcomes?)	1	1	1 ^b^
17	¿Conoce los eventos adversos que han ocurrido como resultado del comportamiento disruptivo? (Are you aware of any specific adverse events that did occur as a result of disruptive behavior?)	0.81	0.81	1 ^b^
17.1	Si la respuesta es sí, por favor descríbalos: (If yes, please describe)	0.90	0.90	0.500 ^b^
17.2	¿Esto se podría haber prevenido? (Could this have been prevented?)	1	1	100 ^c^
17.3	Si la respuesta es sí, por favor descríbalo: (If yes, please describe)	0.90	0.90	100 ^c^
18	¿Existe algún protocolo para actuar frente al comportamiento disruptivo en su hospital? (Is there a code of conduct or policy for the handling of disruptive/abusive behavior at your hospital?)	1	1	100 ^c^
18.1	Si la respuesta es sí, por favor explíquelo: (If yes, please explain)	0.81	0.81	100 ^c^
18.2	¿El protocolo es efectivo? (Is the plan effective?)	0.90	0.90	100 ^c^
18.3	Por favor explíquelo: (Please explain)	0.81	0.81	
19	Si conoce médicos que han sido asesorados debido a su comportamiento, en una escala de 1-10 siendo 10 completamente satisfecho, califique el éxito del proceso. (If you know of physicians who have been counseled about his or her behavior, on a scale of 1-10 with 10 being completely satisfied, rate the success of this process.)	0.90	0.90	0.914 ^a^
19.1	Si conoce enfermeros/as que han sido asesorados/as debido su comportamiento, en una escala de 1–10 siendo 10 completamente satisfecho, califique el éxito del proceso. (If you know of nurses who have been counseled about his or her behavior, on a scale of 1–10 with 10 being completely satisfied, rate the success of this process.)	0.90	0.90	0.927 ^a^
20	¿Existe un servicio de registro no punitivo para aquellos que presencian/experimentan comportamiento disruptivo? (Is there a non-punitive reporting environment for those who witness/experience disruptive behavior?)	0.81	1	100 ^c^
21	¿Existe alguna barrera u obstáculo para notificar un comportamiento disruptivo? (Are there any barriers or resistance to the reporting of disruptive behavior?)	0.81	1	100 ^c^
	**Content Validity Index (CVI) of the set of items in the Scale**	0.89	0.94	

S.R. Statistics Reliability; ^a^ Intraclass Correlation Coefficient; ^b^ Kappa Index; ^c^ General Agreement Index.

## Data Availability

The data presented in this study are available on request from the corresponding author.

## Appendix A

**Table A1:** Cuestionario adaptado—Versión español/España.

Versión Original	Versión Adaptada y Validada
Title: Nurse–Physician Relationship Survey: Impact of Disruptive Behavior on Patient Care (versión original)	Título: Escala sobre la relación Enfermero/a-médico: impacto del comportamiento disruptivo en la atención al paciente
“Disruptive behavior” is defined as any inappropriate behavior, confrontation or conflict ranging from verbal abuse to physical or sexual harassment. One potential consequence of disruptive behavior is its effect on collaboration and communication between physicians and nurses that may result in an adverse effect on patient care. The current survey is designed to assess the potential impact of disruptive behavior on adverse events, medical errors, patient safety, quality and other outcomes of care.	El “Comportamiento disruptivo” se define como cualquier comportamiento inapropiado, enfrentamiento o conflicto que puede ir desde el abuso verbal hasta el físico o acoso sexual. Una de las potenciales consecuencias del comportamiento disruptivo son sus efectos en la colaboración y comunicación entre los médicos y enfermeras que pueden conllevar resultados negativos en la atención al paciente. El presente cuestionario ha sido diseñado para identificar el potencial impacto del comportamiento disruptivo en los eventos adversos, errores médicos, seguridad de paciente, calidad y otros aspectos relacionados con la atención.
Please choose only one answer for each question unless otherwise stated. If you are completing the survey electronically, you can double click on any box and a prompt will appear. Under default value, click on “checked” and an X will appear in the box.	Por favor, escoja solo una de las respuestas por cada pregunta, salvo que se indique lo contrario. En caso de contestar el cuestionario de forma electrónica, puede hacer doble clic en cualquier casilla y aparecerá un aviso de confirmación. Debajo de cada valor, haga clic sobre la respuesta y una X aparecerá en la casilla.
*(Survey Demographics: Please choose one in each category)*	(Variables sociodemográficas: Por favor, seleccione una por cada categoría)
*Clinical is defined as 50% or more of time spent with clinical duties*	Clínico: se define a los profesionales que pasan el 50% o más de su jornada en tareas clínicas
*Executive is defined as 50% or more of time spent with administrative duties*	Administrativo: se define a los profesionales que pasan el 50% o más de su jornada en tareas administrativas
*Title*	Cargo
Physician (Clinical)	Médico (Clínico)
Physician (Executive)	Médico (Administrativo/gestión)
RN (Executive)	Enfermero/a (Administrativo/gestión)
RN (Clinical)	Enfermero/a (Clínico)
Administration	Administración
Other ____________	Otros
*Service*	Servicio
Medical Service	Centro de salud
Emergency Department	Urgencias
Intensive Care	Cuidados intensivos
Surgical Services	Cirugía
Other __________	Otros
*Demographics*	Sociodemográfica
19 Years or Younger	19 años o menos
20–29 Years	20–29 años
30–39 Years	30–39 años
40–49 Years	40–49 años
50–59 Years	50–59 años
60 Years or Older	60 años o más
Male	Hombre
Female	Mujer
(1) On a scale of 1–10 with 10 being the most positive, how would you describe the overall atmosphere of nurse–physician relationships at your hospital?	(1) En una escala del 1 al 10, siendo 10 la más positiva, ¿cómo describiría el ambiente de la relación Enfermera–Médico en su hospital?
Very negative	Muy negativa
Barely Positive	Poco Positiva
Somewhat Positive	Algo positiva
Mostly Positive	Bastante positiva
Very Positive	Muy positiva
(2) Have you ever witnessed disruptive behavior from a physician at your hospital?	(2) ¿Alguna vez ha presenciado comportamiento disruptivo por parte de un/a médico de su hospital?
(3) Have you ever witnessed disruptive behavior from a nurse at your hospital?	(3) ¿Alguna vez ha presenciado comportamiento disruptivo por parte de un/a enfermero/a de su hospital?
(4) Are there any particular settings where disruptive behavior is most prevalent? (Check all that apply)	(4) ¿Existe alguna unidad en la que el comportamiento disruptivo es más prevalente? (marque todas las áreas)
ICU	UCI
OR	Quirófanos
ED	Urgencia
OB	Obstetricia
Med unit	Med. general
surg. Unit	Cirugía
SNF	Enfermería
Other	Otros
(5) Are there any particular specialties where disruptive events occur most often? (Check all that apply)	(5) ¿Existe alguna especialidad donde se produzcan comportamientos disruptivos de forma frecuente? (Seleccione todas las especialidades)
General Surgery	Cirugía General
Cardiac Surgery	Cirugía Cardiaca
Cardiology	Cardiología
Orthopedics	Ortopédica
Neurosurgery	Neurocirugía
Anesthesia	Anestesia
OB/Gyn	Obstetricia/Ginecología
Other	Otros
(6) What percentage of physicians would you say exhibit disruptive behavior at your hospital?	(6) ¿Qué porcentaje de médicos diría que muestran comportamiento disruptivo en su hospital?
None	Cero
More than 10%	Más del 10%
(7) What percentage of nurses would you say exhibit disruptive behavior at your hospital?	(7) ¿Qué porcentaje de enfermeros/as diría que muestran comportamiento disruptivo en su hospital?
None	Cero
More than 10%	Más del 10%
(8) How often does physician disruptive behavior occur at your hospital?	(8) ¿Con qué frecuencia los médicos presentan comportamientos disruptivos en su hospital?
Daily	Diariamente
Weekly	Semanalmente
1–2 Time/Month	1–2 veces al mes
1–5 Times/Year	1–5 veces al año
Never	Nunca
(9) How often does nurse disruptive behavior occur at your hospital?	(9) ¿Con qué frecuencia los enfermeros/as presentan comportamientos disruptivos en su hospital?
Daily	Diariamente
Weekly	Semanalmente
1–2 Time/Month	1–2 veces al mes
1–5 Times/Year	1–5 veces al año
Never	Nunca
(10) On a scale of 1–10 with 10 being the most serious, how serious of an issue is physician disruptive behavior at your hospital?	(10) En una escala del 1–10 siendo 10 los casos más graves, ¿Cómo de graves son los problemas causados por el comportamiento disruptivo de los médicos en su hospital?
Not Serious	Nada graves
Minimally Serious	poco graves
Somewhat Serious	Algo graves
Mostly Serious	Bastante graves
Very Serious	Muy graves
(11) How serious of an issue is nurse disruptive behavior in your hospital?	(11) ¿Cómo de graves son los problemas causados por el comportamiento disruptivo de enfermeros/as en su hospital?
Not Serious	Nada graves
Minimally Serious	Mínimamente graves
Somewhat Serious	Algo graves
Mostly Serious	Bastante graves
Very Serious	Muy graves
(12) From your perspective, do you think that disruptive behavior can potentially have a negative effect on patient outcomes?	(12) Desde su punto de vista, ¿Cree que el comportamiento disruptivo puede tener efectos potencialmente negativos en la atención al paciente?
(13) How often do you think disruptive behavior results in the following? (never-rarely-sometimes-frequent-constant)	(13) ¿Con qué frecuencia cree que comportamiento disruptivo influye en los siguientes aspectos? (nunca-en raras veces-algunas veces-frecuentemente-constantemente)
Stress	Estrés
Frustration	Frustración
Loss of concentration	Pérdida de concentración
Reduced team collaboration	Trabajo en equipo reducido
Reduced information transfer	Información transmitida reducida
Reduced communication	Comunicación reducida
Impaired RN-MD relations	Problemas en la relación Enfermera—Médico
(14) How often do you think there is a link between disruptive behavior and the following? (never-rarely-sometimes-frequent-constant)	(14) ¿Con que frecuencia considera que existe una relación entre el comportamiento disruptivo y los siguientes aspectos? (nunca-en raras veces-algunas veces-frecuentemente-constantemente)
Adverse Events	Eventos Adversos
Errors	Errores
Patient safety	Seguridad del paciente
Quality of care	Calidad de la atención
Patient mortality	Mortalidad del paciente
Nurse satisfaction	Satisfacción de los/as enfermeros/as
Physician satisfaction	Satisfacción de los médicos
Patient satisfaction	Satisfacción del paciente
(15) Are you aware of any potential adverse events that could have occurred from disruptive behavior?	(15) ¿Tiene conocimiento de cualquier evento adverso potencial que pudiera haber ocurrido debido un comportamiento disruptivo?
(16) If yes, how serious an impact do you think this could have had on patient outcomes?	(16) Si la respuesta es sí, ¿Cómo de grave hubiera sido el impacto en los pacientes?
Not Serious	Nada grave
Somewhat Serious	Algo grave
Serious	Grave
Very Serious	Muy grave
Extremely Serious	Extremadamente grave
(17) Are you aware of any specific adverse events that did occur as a result of disruptive behavior?	(17. ¿Conoce los eventos adversos que han ocurrido como resultado del comportamiento disruptivo?
(17.1) If yes, please describe:	(17.1) Si la respuesta es sí, por favor descríbalos:
(17.2) Could this have been prevented?	(17.2) ¿Este se podría haber prevenido?
(17.3) If yes, please describe:	(17.3) Si la respuesta es sí, por favor descríbalo:
(18) Is there a code of conduct or policy for the handling of disruptive/ abusive behavior at your hospital?	(18) ¿Existe algún protocolo para actuar frente al comportamiento disruptivo en su hospital?
(18.1) If yes, please explain:	(18.1) Si la respuesta es sí, por favor explíquelo:
(18.2) Is the plan effective?	(18.2) ¿El protocolo es efectivo?
(18.3) Please explain:	(18.3) Por favor explíquelo:
(19) If you know of physicians who have been counseled about his or her behavior, on a scale of 1–10 with 10 being completely satisfied, rate the success of this process.	(19) Si conoce médicos que han sido asesorados debido a su comportamiento, en una escala de 1–10 siendo 10 completamente satisfecho, califique el éxito del proceso
Not Satisfied	Nada satisfecho
Minimally Satisfied	Poco satisfecho
Somewhat Satisfied	Algo satisfecho
Mostly Satisfied	Muy Satisfecho
Completely Satisfied	Completamente Satisfecho
(a) If you know of nurses who have been counseled about his or her behavior, on a scale of 1–10 with 10 being completely satisfied, rate the success of this process.	(a) Si conoce enfermeros/as que han sido asesorados/as sobre su comportamiento, en una escala de 1-10 siendo 10 completamente satisfecho, califique el éxito del proceso.
Not Satisfied	Nada satisfecho
Minimally Satisfied	Poco satisfecho
Somewhat Satisfied	Algo satisfecho
Mostly Satisfied	Muy Satisfecho
Completely Satisfied	Completamente Satisfecho
(20) Is there a non-punitive reporting environment for those who witness/experience disruptive behavior?	(20) ¿Existe un servicio de registro para aquellos que presencian/experimentan comportamiento disruptivo?
(21) Are there any barriers or resistance to the reporting of disruptive behavior? (Check all that apply):	(21) ¿Existe alguna barrera u obstáculo para notificar un comportamiento disruptivo? (Seleccione todos los aspectos):
Fear of Retaliation	Miedo a las represalias
Lack of Confidentiality	Falta de confidencialidad
The feeling that “Nothing ever changes”	La sensación de que “nada cambiara nunca”
No feedback of results	No existe respuesta o resultados
Other	Otros
Please explain:	Por favor explíquelos:
Additional Comments (use additional space as necessary)	Comentarios adicionales (utilice el espacio adicional en caso de ser necesario)
Your Title (optional)	Su cargo en el trabajo (opcional)
